# Quantifying the parallel mediation influence of body mass index and depression on physical activity and cognitive function among 3,611 Chinese older adults

**DOI:** 10.3389/fnagi.2022.977321

**Published:** 2022-09-07

**Authors:** Ji Liu, Faying Qiang, Jingxia Dang, Qiao Yi Chen

**Affiliations:** ^1^Faculty of Education, Shaanxi Normal University, Xi’an, China; ^2^Department of Neurology, The First Affiliated Hospital of Xi’an Jiaotong University, Xi’an, China; ^3^School of Basic Medical Sciences, Xi’an Jiaotong University, Xi’an, China

**Keywords:** aging, physical activity, cognitive function, BMI—body mass index, depression

## Abstract

**Background:**

Engagement in physically active lifestyles brings multidimensional health benefits including better cognitive function. While prior studies examined the link between physical activity and cognitive function, a remaining unanswered question is what modifiable factors channel such effects.

**Objective:**

This study investigates the extent to which subject’s body mass index (BMI) and depression mediate the link between physical activity and cognitive function among older adults in China.

**Methods:**

This study builds a parallel structural equation model utilizing the 2013–2018 China Health and Retirement Longitudinal Study (CHARLS) dataset. We screened a total of 14,724 subjects, among which 3,611 subjects met the inclusion criteria. Physical activity, depression, and cognitive function are measured using the International Physical Activity Questionnaire (IPAQ), Center for Epidemiological Research Depression Scale (CES-D), and Mini-Mental State Examination (MMSE) instruments.

**Results:**

Parallel mediation analyses indicate that depression significantly mediates the link between physical activity and cognitive function (std. β = 0.023, *p*-value = 0.010), while no significant mediation was observed *via* BMI (std. β = 0.005, *p*-value = 0.155). Findings also show that physical activity is positively associated with cognitive function (std. β = 0.104, *p*-value = 0.004), whereas physical activity is inversely associated with BMI (std. β = –0.072, *p*-value = 0.045). Both BMI (std. β = –0.071, *p*-value = 0.042) and depression (std. β = –0.199, *p*-value = 0.001) are negatively associated with cognitive function.

**Conclusion:**

This study quantifies the positive association between physical activity and cognitive function in older Chinese adults, and uncovers a significant mediation channel occurring through depression. From a clinical perspective, physical behavioral modifications can lead to linked improvements in both mental and cognitive wellbeing for older adults.

## Introduction

The World Health Organization [WHO] (2021) estimates that by 2050, the world’s population of people over 60 years old will double and that for people over 80 years old will triple, bringing about seismic demographic transition. China has the world’s largest population, and one of the fastest growing aging population; it is projected that by 2040, proportion of people in China over 60 years old is expected to reach 28%, approximately 6% higher than the estimated global average (Bao et al., 2022). Consequentially, aging is associated with a naturally irreversible deterioration of one’s physical and mental capacity over time, leading to a range of age-related diseases including mild cognitive impairment, depression, hypertension, diabetes, etc. (Titorenko, 2019).

The risk of cognitive decline and impairment among older adults is rapidly rising, and have become a major public health challenge globally (Ren et al., 2022). According to the World Health Organization [WHO] (2020), about 55 million older adults worldwide are living with dementia, among them 15 million reside in China (Jia et al., 2020). Dementia is characterized by the loss of foundational cognitive function such as thinking, reasoning, and memory, which impede daily life activities and are found to increase risks of shorter life expectancy (National Institute on Aging, 2021). While there is no definitive cure for cognitive decline, modifiable risk factors such as behavioral and psychological conditions show promise in mitigating its adverse health consequences (Northey et al., 2018).

In existing studies, physical activity is considered one effective therapeutic and prophylactic measure for combating cognitive decline (Krivanek et al., 2021). Recent evidence has emphasized that physical activity serves as an important neuroprotective modulator for improving overall cognition function, especially in preventing cognitive impairment-related diseases and enhancing brain function in older populations (Jia et al., 2019). Boosting physical activeness can facilitate glucose control, metabolic response, cardiovascular function, and general physical health among older adults (Ruegsegger and Booth, 2018). In addition, physical activity stimulates cerebral cortex activation, accelerates the release of key neurochemicals, and can reduce inflammatory response (Cirillo, 2021). Importantly, the cerebral cortex is involved in high-level brain processes including language, sensory, memory, and reasoning (Hu et al., 2019). Release of key neurochemicals such as brain-derived neurotrophic factor and insulin growth factor can improve neuroplasticity in the brain (Gheysen et al., 2018), while reduction in levels of inflammatory markers such as C-reactive protein and interleu-kin-6 can prevent deterioration of brain function associated with old age (Morgan et al., 2014).

In conceptual terms, improvements in body fitness and mental wellbeing has been identified as two key channels through which physical activity influence cognitive function. On the one hand, physical activity has been shown to exert protective effect on cognitive function *via* controlling weight gain (O’Brien et al., 2017). Prior studies suggest that encouraging long-term engagement in physical activities among older adults can benefit the cardiopulmonary system for more effective oxygen and nutrient distribution, leading to higher metabolic rates to better regulate glucose and fatty acid metabolism, thereby reducing risk for cognitive impairment as a consequence of obesity (McPhee et al., 2016). Relatedly, weight gain and obesity have been found to be associated with detrimental effects on brain integrity and function in older adults, as higher body mass index (BMI) is often associated with increased risks for cardiovascular disease and metabolic disorders (Hou et al., 2019). Moreover, higher BMI is also correlated with worsened learning, memory and executive function, which are strong predictors of cognitive decline and dementia in later life (Zhou et al., 2010).

On the other hand, mental wellbeing has been proposed as a crucial channel through which physical activity can improve cognitive function among older adults (Liu and Qiang, 2022). While there still exists some debate regarding the bi-directional influence of depression and cognitive decline since depressive symptoms are often found to clinically accompany dementia, longitudinal studies have identified links between depressive episodes and dementia risks (Singh-Manoux et al., 2017). Studies have shown that people suffering from depression are more vulnerable to cognitive impairment due to elevated levels of stress hormones, neuronal growth factors, and hippocampal volume (Reppermund et al., 2007). More recent studies have suggested that physical activity can impart positive influence on the depression-cognition relationship by promoting social interaction and improving psychosocial health (Fu et al., 2018), and through delaying release of stress hormones (Saeed et al., 2019).

**FIGURE 1:**
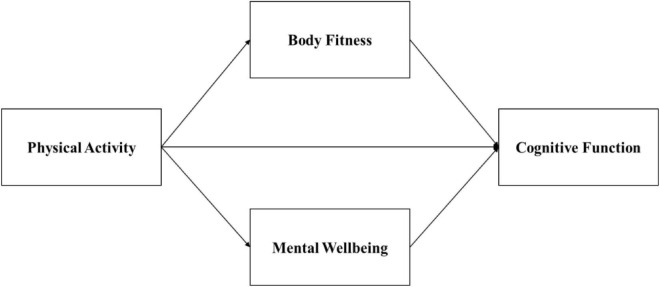
Path diagram of the structural mediation model.

While prior studies provide insight into the influence of physical activity on cognitive function, a remaining underexplored question is whether such relationships are mediated by body fitness or mental wellbeing. This present study aims to fill this void in the existing literature by hypothesizing direct and indirect influence of physical activity on cognitive function as operating *via* two parallel mediators: body fitness and mental wellbeing (Figure 1). The hypothesized model is empirically tested utilizing the structural equation modeling (SEM) technique.

## Materials and methods

### Study sample

**FIGURE 2:**
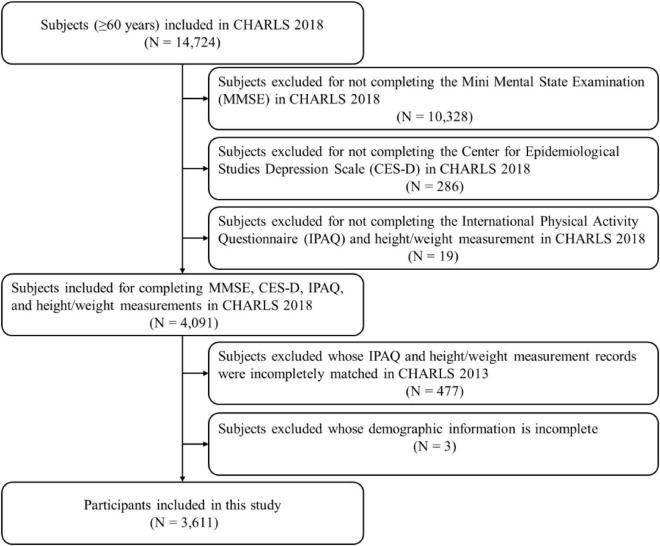
Flowchart of subject inclusion.

This study uses the two most recent waves of the China Health and Retirement Longitudinal Study (CHARLS) dataset, which is derived from a representative national longitudinal investigation of individuals over the age of 45 years-old in China. To ensure sample representativeness, 17,708 participants from 28 provinces, including 150 counties and 450 villages were selected for the 2011 baseline survey, and participants were reassessed in 2013 and 2018 (Zhao et al., 2020). We screened a total of 14,724 subjects over the age of 60 years-old, who had been interviewed in CHARLS 2018 (Figure 2), and found matching records of 3,611 subjects in CHARLS 2013, whom were included in the present study.

Research ethics approval for the CHARLS study was granted by the Institutional Review Board at Peking University (IRB00001052-11015).

### Physical activity

An abbreviated International Physical Activity Questionnaire (IPAQ) form, which is an internationally recognized instrument for soliciting and measuring physical activity, is used (Zhao et al., 2020). Information on subject’s engagement in light physical activity (LPA), moderate physical activity (MPA), and vigorous physical activity (VPA) is collected. Operationally, LPA is defined as activities consisting of metabolic equivalent task (MET) values of less than 3.3; MPA is defined as activities with MET values of approximately 4.0; VPA is defined as activities having MET values of approximately 8.0. According to the IPAQ scoring protocol, a standardized index for subject’s physical activity is computed by weighing LPA, MPA, and VPA based on their respective MET values (Tomioka et al., 2011), using the Equation below:


$$MET_{it}=3.3*LPA_{it}+4*MPA_{it}+8*VPA_{it}$$

MET score is calculated for each subject *i* at time *t*, as a weighted sum of the total number of MET-minutes per week reported by the subjects who participated in LPA, MPA, and VPA. In order to assess relative change in physical activity for each between 2013 and 2018, a relative measure of MET change *MET*_*it*_/*MET*_*i*(*t*−1)_, is calculated for each subject by taking the ratio of their MET scores in 2018 and in 2013. A rate of change value greater than 1 reflects higher MET in 2018 than in 2013, and vice versa should the value be lesser than 1.

### Body mass index

Body fitness is operationally measured using BMI, which is a composite index of weight-for-height measurements that is widely used to classify overweight and obesity. According to the World Health Organizations [WHO] (2021), BMI is computed from self-reported weight in kilograms divided by the square of height in meters (kg/m^2^). Prior studies have highlighted methodological limitations using only one BMI measure, since having the same BMI may imply drastically different BMI due to different antecedent measures (Hernan and Taubman, 2008). Therefore, a relative measure of BMI change *BMI*_*it*_/*BMI*_*i*(*t*−1)_ is calculated for each subject by taking the ratio of their BMI measurements in 2018 and in 2013. A rate of change value greater than 1 reflects higher BMI in 2018 than in 2013, and vice versa should the value be lesser than 1.

### Depression

Mental wellbeing is assessed using the 10-item Center for Epidemiological Research Depression Scale (CES-D) instrument, which has been validated for depression diagnosis (Andresen et al., 1994). The CES-D utilizes four response options for each item, including “rarely (< 1 day/week)”; “some days (1–2 days/week)”; “occasionally (3–4 days/week)”; and “most (5–7 days/week)” to assess prevalence of depressive symptoms (Radloff, 1977). In this study, CES-D items are used as measurements for the latent construct “Depression,” which is a widely adopted practice in existing studies (Kozlov et al., 2020).

### Cognitive function

Cognitive function is evaluated using the Mini-Mental State Examination (MMSE) instrument, which is a widely adopted cognitive assessment tool used in clinical diagnosis for dementia and Alzheimer’s disease (Crum et al., 1993). The translated and locally adapted version of MMSE has been extensively used in China for clinical diagnosis of cognitive impairment (Yi and Vaupel, 2002), and is administered to subjects using their preferred dialect in the CHARLS study. The test instruments included immediate and delayed word recall, word recognition, animal naming, symbol digit modalities test, digit span backward test, immediate, and delayed logical memory, immediate constructional praxis, number series, and trail-making tests. Following MMSE scoring protocol, we compute a composite score with a maximum possible outcome of 30 points (Zhou et al., 2021).

### Statistical analysis

In this study, we conduct two inter-related analyses using STATA version 15.1 (Stata, StataCorp LLC, College Station, TX, United States) software. In the first analysis, key descriptive information is reported for bivariate statistical tests. The critical *p*-value was set at 0.05. In the second analysis, we build a SEM to examine the interlinked relationship between physical activity and cognitive function, and fit two parallel mediation pathways in evaluating the extent to which BMI and depression influence this link. Several goodness-of-fit indices are reported in evaluating how well the structural model fit the data, including the comparative fit index (CFI), Tucker-Lewis index (TLI), root mean square error approximation (RMSEA), and the standardized root mean square residual (SRMR). It should be emphasized that in this study, the natural direct pathway herein reflects the net association between change in physical activity and cognitive function, conditional on a set level of the proposed mediators: BMI and depression, while the natural indirect pathway refers to the net association between physical activity and cognitive function through pathways mediated by BMI and depression.

## Results

### Descriptive statistics

Table 1 reports the descriptive statistics of subjects and results of bivariate statistical tests. A total of 3,611 older adults are included in this study. The subjects’ mean age in 2018 was 68.513 years old (*SD* = 7.082), and the average MET rate of change and BMI rate of change is 2.500 (*SD* = 8.342) and 0.999 (*SD* = 0.098), respectively. Bivariate correlation is reported in the final two columns in Table 1, such that there is no statistically significant relationship MET rate of change and age (*p*-value = 0.837), nor with BMI rate of change (*p*-value = 0.500). MET rate of change is positive and significantly correlated with CES-D score (*p*-value = 0.001) and MMSE score (*p*-value = 0.001) in 2018. Independent t- test results are reported in the final two columns of rows 7–15 in Table 1. There is no statistical difference in MET rate of by sex (*p*-value = 0.144), residence location (*p*-value = 0.174), or marital status (*p*-value = 0.846).

**TABLE 1:** Subject characteristics.

	Mean (*N*)	*SD* (%)	Association with MET rate of change (2018/2013)	
			Pearson’s R (T-statistic)	*P*-value
Age (2018)	68.513	7.082	0.004	0.837
MET rate of change (2018/2013)	2.500	8.342	1.000	–
BMI rate of change (2018/2013)	0.999	0.098	–0.018	0.500
CES-D score (2018)	7.834	5.773	–0.044	0.001*
MMSE score (2018)	22.938	5.395	0.066	0.001*
Sex				
Male	1,726	47.80	–1.461	0.144
Female	1,885	52.20		
Residence location (2013)				
Urban	747	20.69	–1.361	0.174
Rural	2,864	79.31		
Marital status (2013)				
Married	3,112	86.18	–0.195	0.846
Otherwise	499	13.82		

*Denotes p-value < 0.05.

### Reliability and convergent validity of CES-D instruments

Table 2 indicate reliability and convergent validity results of the latent construct “depression.” Measurement components show relatively high Cronbach alpha of 0.782, factor loadings that range between 0.499 and 0.777, Kaiser-Meyer-Olkin measure of sampling adequacy is 0.859, Bartlett’s test-of-sphericity statistic is 7062.490 (*p*-value = 0.001), all of which indicate good within-construct reliability and convergent validity.

**TABLE 2:** Reliability and convergent validity of CES-D.

Items		Factor loadings
1	I was bothered by things that don’t usually bother me	0.696
2	I had trouble keeping my mind on what I was doing	0.680
3	I felt depressed	0.777
4	I felt everything I did was an effort	0.693
5	I felt hopeful about the future (reversed)	0.476
6	I felt fearful	0.583
7	My sleep was restless	0.499
8	I was happy (reversed)	0.479
9	I felt lonely	0.659
10	I could not “get going”	0.650

Cronbach alpha is 0.782, Kaiser-Meyer-Olkin measure of sampling adequacy is 0.859, Bartlett’s test-of-sphericity statistic is 7062.490 (p-value = 0.001).

### Parallel mediation analysis

**FIGURE 3:**
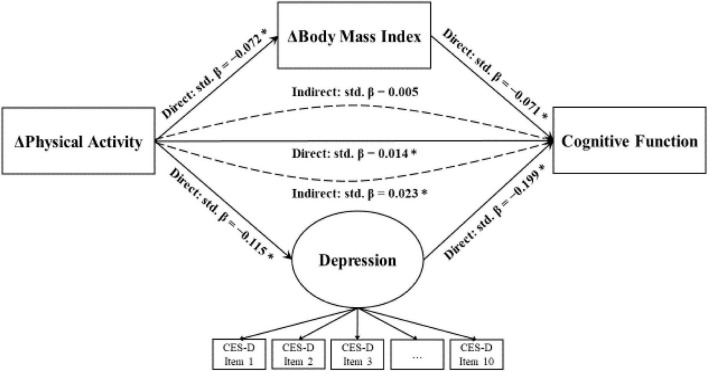
Standardized solution of the structural mediation model. *p-value < 0.05.

Table 3 and Figure 3 show point estimates for standardized direct and indirect effects. For model goodness-of-fit, several key measures are jointly evaluated, including CFI = 0.955, TLI = 0.945, RMSEA = 0.041, and SRMR = 0.040. Overall, CFI and TLI statistics are above the recommended threshold of 0.90, while for RMSEA and SRMR statistics are below recommended upper-bound limits of 0.06 and 0.08, respectively, which jointly implies good model fit (Hooper et al., 2008).

**TABLE 3:** Parallel mediation analysis results.

Pathways		Standardized coefficient	S.E.	*Z*	*P*-value	95% CI
Direct						
	PA → CF	0.104	0.035	2.910	0.004*	(0.033, 0.171)
	PA → BMI	–0.072	0.036	–2.000	0.045*	(–0.142, –0.002)
	PA → DEP	–0.115	0.039	–2.970	0.003*	(–0.190, –0.039)
	BMI →CF	–0.071	0.035	–2.030	0.042*	(–0.140, –0.002)
	DEP → CF	–0.199	0.038	–5.260	0.001*	(–0.273, –0.125)
Indirect						
	PA → BMI → CF	0.005	0.004	1.424	0.155	(–0.002, 0.012)
	PA → DEP → CF	0.023	0.009	2.573	0.010*	(0.005, 0.042)

PA, physical activity; CF, cognitive function; DEP, depression; BMI, Body Mass Index. *Denotes p-value < 0.05. Model fit indices are CFI = 0.955, TLI = 0.945, RMSEA = 0.041, and SRMR = 0.040.

For direct pathways, standardized coefficient (std. β) results indicate that physical activity, as conceptualized as the rate change of MET score between 2013 and 2018, is positively and significantly associated with cognitive function (std. β = 0.104, *p*-value = 0.004), whereas physical activity is negatively and significantly associated with BMI rate of change (std. β = –0.072, *p*-value = 0.045) and the latent construct “depression” (std. β = –0.115, *p*-value = 0.003). To add, model results suggest that BMI rate of change (std. β = –0.071, *p*-value = 0.042) and depression (std. β = –0.199, *p*-value = 0.001) are negatively associated with cognitive function.

For indirect pathways, we conduct two independent mediation tests using Delta, Sobel, and Monte Carlo statistical tools. Results jointly show that there only exits one unique indirect channel through which depression (std. β = 0.023, *p*-value = 0.010) significantly mediates the influence of physical activity on cognitive function. An indirect channel does not exist for BMI (std. β = 0.005, *p*-value = 0.155). The size of the indirect effect operating through depression is estimated to be 22.12% of that direct effect (0.023/0.104), or 18.11% of the total effect (0.003/0.037).

## Discussion

This study investigates the longitudinal relationship between physical activity, BMI, depression, and cognitive function in 3,611 Chinese subjects over the age of 60 years old. Results indicate that increased engagement in physical activity over a course of 5 years was conducive to reducing BMI increase (std. β = –0.072, *p*-value = 0.045), while higher rate of change in BMI is correlated with worsened performance in cognitive function in late adult life (std. β = –0.071, *p*-value = 0.042). Consistent with existing studies in aging research, these results imply that physical activity positively affects body fitness, and that decline in body fitness is predictive of decline in cognitive function (Liang et al., 2022). Relatedly, physical inactivity has been identified as a critical risk factor accelerating age-related deterioration in physiological function and onset of chronic disorders such as obesity, whereas active participation in physical activities is considered an effective strategy for preventing weight gain and slowing cognitive decline (Pethick and Piasecki, 2022). Behavioral risk factors leading to increase in BMI among older adults could account for the elevated cognitive-related burden (Livingston et al., 2020), since late adulthood obesity is considered one of the primary risk factors associated with dementia and Alzheimer’s disease (Pitrou et al., 2022). In addition, results also suggest that encouraging active life styles, such as those rich in physical activity engagement, could be effective behavioral strategies that create an age-buffering effect in mitigating adverse consequences of aging, since enhanced physical activity helps delay adverse change in brain structure and volume, such as volume of hippocampus, white matter, and basal ganglia, which actively promotes brain response and nervous system activity (Ingold et al., 2020).

One novel finding in this study is that depression provided channeling effect through which physical activity can have a significant influence on cognitive function, accounting for as much as 22.12% of the observed direct effect. The present study contributes new evidence to a growing body of aging research that highlights the key mediator role of depression on the link between late adulthood lifestyle and cognition (Marashi et al., 2021). Prior research indicates that executive control supported by the frontal cortex is a key cognitive mechanism associated with behavioral responses to stress and anxiety, and that poor state of psychosocial wellbeing may further hinder the limited capacity for cognitive processing (Bauermeister and Bunce, 2015). Recent aging neuroscience research has found that depressive behaviors are associated with worsened neuroplasticity, including neuronal atrophy and synaptic loss in the medial prefrontal cortex and hippocampus (Price and Duman, 2020). At the same time, frequent physical activity may delay cognitive decline *via* offering psychosocial avenues for improving state of mental wellbeing (Liu et al., 2022). Active participation in physical activities can help foster healthy lifestyles, improve social relationships, promote life satisfaction, and enhance self-regulation, which are favorable determinants of cognitive function in late adulthood (Ouanes et al., 2017). In biophysiology terms, prior studies show that active life styles increase endorphin levels, promote mitochondrial function, increase mitochondrial generation and neurotransmitter production, which are directly associated with better cognitive function (Mikkelsen et al., 2017). Taken altogether, emerging evidence seems to corroborate that the complex inner-structure of the neurocognitive system is highly inter-linked (Chen et al., 2021), such that both physiological and psychological factors affect the brain’s capacity to cognitively make-meaning, respond, and adapt (Moda-Sava et al., 2019).

Finally, while results illuminate the association between body fitness and cognitive function, we do not find conclusive evidence in support of identifying an indirect channel linking physical activity, body fitness, and cognitive function. This result suggests that while physical activity is inversely related to BMI, their links to cognitive function remain independent. Relating these conclusions to prior studies that found weak associations between biomarkers and cognitive impairment (Jensen et al., 2015), there seems to exist a knowledge gap regarding the neuropathologic mechanism of how BMI may play protective roles against cognitive decline. Nonetheless, these gaps generate considerable new interests in motivating future research in aging neuroscience.

## Strengths and limitations

This study has several strengths. First, a large-scale nationally representative 5-year longitudinal dataset was utilized. Second, the study is designed to quantify the influence of modifiable risk factors on cognitive function, utilizing a parallel mediation approach. Third, findings provide new insights on the indirect channeling mechanisms of depression on linking physical activity and cognitive function among older adults. Finally, our use of structural mediation modeling simultaneously analyzes both direct and indirect pathways, and reduces type-I error occurrence as compared to traditional regression-based statistical analyses. For study limitations, the CHARLS study had only included the abbreviated version of the IPAQ CES-D and MMSE instruments, which could potentially limit precise diagnosis, and physical activity measurement precision could be improved in future studies. In addition, since this present study does not randomize individuals to different levels of physical activity, the analysis is limited in its ability to draw causal conclusions.

## Conclusion

In conclusion, this study highlights a positive association between physical activity and cognitive function, as mediated by depression in older Chinese adults. Furthermore, long-term engagement in physical activities was conducive to limiting BMI increase, which was positively correlated with rate of cognitive decline, although there is no evidence of an indirect channel linking physical activity, BMI, and cognitive function.

## Data availability statement

Publicly available datasets were analyzed in this study. This data can be found here: http://charls.pku.edu.cn/en/.

## Ethics statement

The studies involving human participants were reviewed and approved by the Institutional Review Board at Peking University. The patients/participants provided their written informed consent to participate in this study.

## Author contributions

JL and QC conceived, conceptualized, and designed the study and contributed to the statistical analysis. JL, FQ, QC, and JD drafted the article and contributed to the interpretation of results. All authors have revised the manuscript for important intellectual content and have read and agreed to the present version of the manuscript.
